# Mechanisms of Cancer Cell Death: Therapeutic Implications for Pancreatic Ductal Adenocarcinoma

**DOI:** 10.3390/cancers13194834

**Published:** 2021-09-28

**Authors:** Hannah Pook, Siim Pauklin

**Affiliations:** Botnar Research Centre, Nuffield Department of Orthopaedics, Rheumatology and Musculoskeletal Sciences, Old Road, University of Oxford, Oxford OX3 7LD, UK; hannah.pook@sjc.ox.ac.uk

**Keywords:** pancreatic cancer, PDAC, epigenetics, metabolism, cancer stem cells, cancer therapy

## Abstract

**Simple Summary:**

Pancreatic cancer is a disease which is known to be highly deadly, with only 8% of people diagnosed surviving for more than 5 years. Multiple factors contribute to this poor survival rate, including a lack of early symptoms leading to late diagnosis and a high tendency for the cancer to rapidly spread throughout the body. Although there has been a lot of work in this area, we still lack an effective cure for pancreatic cancer. In this review, we aim to outline the latest research on possible treatments, including those which disrupt signalling pathways in the cell, disturb the cancers’ fuel sources, overload cancer cells with mutations, and re-direct the body’s immune system to attack the tumour. We also look at how we could eliminate a particularly aggressive group of cells within the tumour (‘cancer stem cells’) which are thought to be important to pancreatic cancer growth and spread.

**Abstract:**

Pancreatic ductal adenocarcinoma (PDAC) is a type of cancer that is strongly associated with poor prognosis and short median survival times. In stark contrast to the progress seen in other cancer types in recent decades, discoveries of new treatments in PDAC have been few and far between and there has been little improvement in overall survival (OS). The difficulty in treating this disease is multifactorial, contributed to by late presentation, difficult access to primary tumour sites, an ‘immunologically cold’ phenotype, and a strong tendency of recurrence likely driven by cancer stem cell (CSC) populations. Furthermore, apparently contrasting roles of tumour components (such as fibrotic stroma) and intracellular pathways (such as autophagy and TGFβ) have made it difficult to distinguish beneficial from detrimental drug targets. Despite this, progress has been made in the field, including the determination of mFOLFIRINOX as the standard-of-care adjuvant therapy and the discovery of KRAS^G12C^ mutant inhibitors. Moreover, new research, as outlined in this review, has highlighted promising new approaches including the targeting of the tumour microenvironment, enhancement of immunotherapies, epigenetic modulation, and destruction of CSCs.

## 1. Introduction

Pancreatic ductal adenocarcinoma (PDAC) is a type of cancer that arises in the exocrine glands of the pancreas [1] and comprises over 90% of pancreatic malignancies [2]. Currently the 11th most common cancer worldwide [3], PDAC is the seventh leading cause of cancer-related deaths and is on track to move to second place by 2030 [1]. Despite the high prevalence, therapeutic options remain limited, with only modest improvements in overall survival (OS) occurring over the past 50 years [4]. The 5-year overall survival is currently just 8% [5], with median survival time remaining under 1 year even with newer treatments [6], highlighting a clear need for new and effective therapies. Despite extensive research in the area, surgery is still the only curative option, although late presentation and high rates of metastases by the time of detection limit this approach to just 20% of newly diagnosed cases [7]. Even in those suitable for surgery, re-emergence occurs in 90% following the operation [8]. The current outlook for patients with PDAC continues to be poor and highlights a clear need for new therapeutic options.

Cytotoxic chemotherapy remains the mainstay of treatment in PDAC [9], although research has led to optimised combinations to provide improvements in OS. The PRODIGE-24 trial placed modified 5-fluoro-uracil (5-FU), leucovorin, irinotecan, and oxaliplatin (mFOLFIRINOX) as the standard-of-care adjuvant therapy following significantly increased OS in comparison to gemcitabine (54.4 months vs. 35 months) [10], while in patients with advanced-stage disease, combinations such as paclitaxel and gemcitabine or FOLFIRINOX have also elicited modestly improved OS [6,11]. Research aiming to generate new therapies has had a broad focus, likely accounted for by the highly heterogenous nature of PDAC cancers and lack of actionable genetic aberrations. The most common mutation, which occurs in the KRAS proto-oncogene and is seen in >95% of human PDACs [12], is unfortunately largely unactionable. This may be set to change, however, due to the emergence of direct KRAS inhibitors able to target the G12C mutant KRAS variant [13,14]. Although this mutation is present in just 1–4% of PDACs [15], and hence the majority of patients will be unable to benefit from this inhibitor if it becomes clinically available, its development is a major step forward in targeting a protein previously thought to be undruggable. 

Other strategies currently being investigated include pathway inhibition, metabolic targeting, DNA damage, immunotherapeutic agents, epigenetic targeting, tumour microenvironment (TME) modification, and destruction of cancer stem cells (CSCs). Pre-clinical successes have been seen in many of these fields, although, as of yet, they have not translated into clinical practice. Outside of therapeutics themselves, the determination of biomarkers to guide drug selection and improvements in pancreatic cancer screening and detection are areas that will be key in improving patient outcomes.

It is now well established within the field that PDACs can be stratified into different molecular subtypes, which impact OS and therapeutic response [16,17]. This was first noted by Collison in 2011 [18], who defined three subtypes (classical, quasi-mesenchymal, and exocrine like) using gene expression microarray analysis in primary resected samples as well as human and mouse cell lines. Several years later, Moffitt et al. [19] used virtual microdissection to digitally separate tumour and stromal gene expression to specify two tumour-specific and two stromal-specific subtypes, while Bailey et al. [20] carried out an integrated genomic analysis to determine four distinct subtypes, as summarised in Table 1. More recent research has served to validate the existence of such subtypes and highlight the significant overlap between these classifications, indicating similarities between classical and pancreatic progenitor subtypes as well as basal-like and squamous tumours [16]. Given the molecular differences arising between subtypes, for example, elevated GATA6 expression in the classical/pancreatic progenitor subtype [18,19,21], they could prove to be a useful modality for patient stratification to determine personalised medicine regimes and thus improve patient outcomes. However, further work still needs to be carried out to unify current classification methods and produce a single comprehensive grouping strategy. This would facilitate the application of knowledge to both clinical and research purposes, making precision medicine more viable in the context of PDAC going forwards.

## 2. Pathway Inhibition

Work over recent years has attempted to target multiple pathways vital to growth and development in PDAC. This has been extensively reviewed by Stoica (2020) [22] and Nevala-Plagemann (2020) [23], with the main findings summarised in Table 2. As with many other approaches, drugs have often shown little or no benefit in clinical trials despite promising pre-clinical results.

## 3. Metabolic Targeting

**Figure 1:**
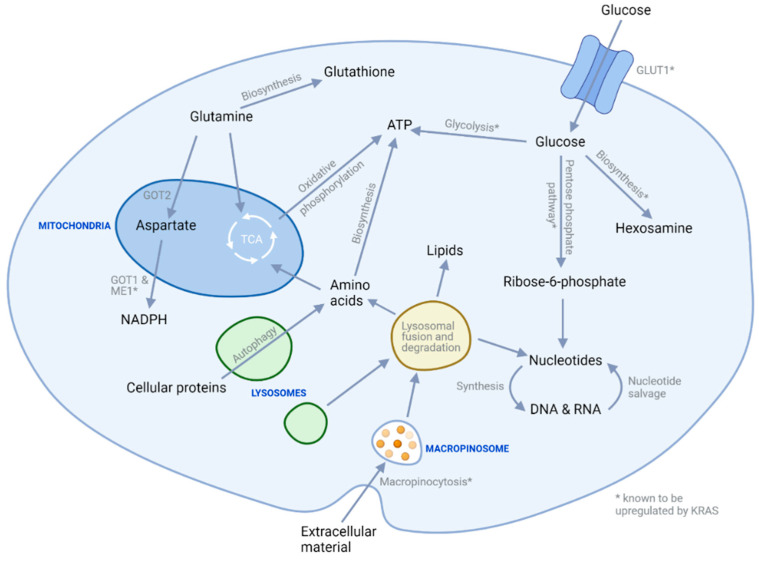
Key metabolic pathways in PDAC cells. Glucose, the intracellular abundance of which is increased by Kras-mediated GLUT1 upregulation, is a key metabolic and biosynthetic fuel usually oxidised in the mitochondria to produce ATP. However, while some mitochondrial oxidation of glucose does occur in cancer cells, proportionally higher levels are diverted from this pathway to be utilised in biosynthetic reactions to produce hexosamine (used in protein glycosylation) and ribose-6-phosphate (used in nucleotide synthesis). Cancer cells also show a greater dependency on glycolytic ATP generation with upregulation of this pathway in PDAC mediated both by oncogenic signalling via the Kras pathway and hypoxic conditions resulting from hypovascularity. Cancer cells also demonstrate an enhanced use of glutamine, which is vital in cellular proliferation due to its use in ATP generation and the production of NADPH and glutathione to maintain cellular redox balance. Nutrient deprivation in PDAC due to hypovascularity also alters cellular metabolism, evoking a requirement for alternative nutrient sources to be found. These include recycling of cellular components through autophagy and nucleotide salvage, as well as scavenging pathways such as macropinocytosis in which large vacuoles non-specifically engulf extracellular space to obtain proteins, lipids, and nucleotides.

As with other cancer types, cellular metabolism in PDAC cells differs from non-cancer cells (Figure 1) [55] and this metabolic dysregulation is an area of interest for therapeutic targeting. Altered metabolism is thought to be due at least in part to KRAS activation [55], and hence it is possible that targeting this pathway may promote beneficial metabolic changes and cancer cell death amongst its other effects within cells. Nonetheless, agents specifically targeting metabolic signalling are of great interest, with a variety of agents being trialled given the metabolic heterogeneity of PDAC [56]. The investigation of metabolic subtypes highlighted groups of cells variably dependent on glycolysis, lipogenesis, and redox pathways, which could enable specific targeting of cellular populations [57]. 

More recently, mitochondrial metabolism has been of interest, with high levels of oxidative phosphorylation (OXPHOS) occurring in a subset of patients [58] despite low oxygen levels within tumours. Subsequent work in PDAC cells and orthotopic mouse models demonstrated that phenformin, a mitochondrial respiratory complex I inhibitor, acted synergistically with gemcitabine to specifically eradicate high OXPHOS cells [58]. Not only did this highlight new therapeutic avenues, it also acts to elucidate a novel patient stratification modality based on tumour metabolism, which could be applied more widely. For example, devimistat is a drug that acts to impair pyruvate dehydrogenase activity in order to selectively inhibit the Krebs cycle in tumour cell mitochondria. It has been hypothesised to work synergistically with cytotoxic agents by decreasing the rate of mitochondrial metabolism and depleting intermediates required for DNA damage repair [23]. In combination with mFOLFIRINOX in a phase I trial involving 20 patients with metastatic PDAC, devimistat was shown to be well tolerated with a response rate of 61% [59]. These promising results allowed devimistat to be granted Orphan Drug Designations for six indications, one of which was pancreatic cancer, and have propelled the drug into a phase III trial (AVENGER 500), which is currently ongoing. Applying the patient stratification model elucidated by Masoud et al. (2020) [58] could highlight patients more likely to benefit from devimistat and thus further improve outcomes. 

Autophagy has been another area of interest in PDAC, being an important energy source that enables cancer cells to survive in the nutrient-deprived environment driven by desmoplasia and poor vascularity. Interestingly, this pathway performs opposing functions throughout the course of tumourigenesis. Initially, autophagy impairs tumourigenesis via the maintenance of cellular homeostasis, but later supports tumour growth by promoting fuel generation via intracellular organelle catabolism [60]. This latter role generated interest in the potential use of hydroxychloroquine, an autophagy inhibitor, as an anti-cancer agent. However, phase II clinical trials in patients with metastatic PDAC unfortunately failed to demonstrate improvements in OS when hydroxychloroquine was used as a single agent [61] or in combination with gemcitabine and nab-paclitaxel [62]. Despite this, hydroxychloroquine continues to be investigated and is currently being administered in a phase I trial in combination with binimetanib, a MAPK inhibitor, in patients with KRAS-mutated metastatic pancreatic cancer [63]. 

Reactive oxygen species (ROS) and antioxidants have similar contrasting roles over the course of PDAC development, with lower levels of ROS promoting tumour initiation at the pre-malignant phase and higher levels then supporting metastatic progression [38]. Attempts to target this pathway have included the inhibition of NRF2 (a master regulator of the cellular stress response), which augmented the effects of gemcitabine in mice [64]. Alternatively targeting this pathway through the inhibition of the ROS sensor PKD1 was able to increase apoptosis in vitro and abrogate tumour growth in vivo, highlighting the potential of this approach [65]. However, caution should be taken when manipulating ROS levels given their contrasting effects dependent on PDAC developmental stage.

## 4. Genome Instability and DNA Damage

**Figure 2:**
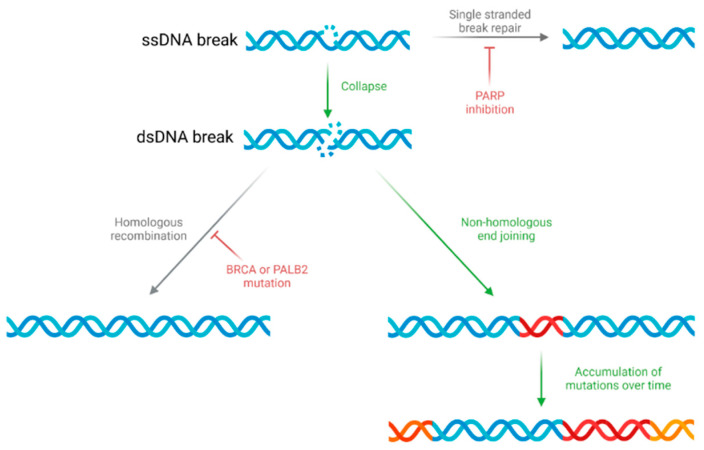
The mechanism of PARP inhibitor-induced cell death. PARP inhibitors prevent cells from carrying out single-stranded DNA (ssDNA) break repair, resulting in the collapse of ssDNA breaks into double-stranded DNA (dsDNA) breaks. In cells with BRCA or PALB2 mutations, homologous recombination cannot occur and hence dsDNA breaks must be repaired through the more error-prone mechanism of non-homologous end-joining. This results in mutations (red), which accumulate over time to induce synthetic lethality.

Eliciting DNA damage in order to promote cellular senescence has proved to be a successful approach in multiple cancer types. Most notably, the use of PARP inhibitors in BRCA mutated breast cancers has been a major step forward [66]. PARP enzymes are vital in single-stranded DNA break repair and in cells already unable to carry out homologous recombination to repair double stranded breaks (as in BRCA or PALB2 mutated cancers) PARP inhibition is able to increase genetic instability to evoke synthetic lethality [67,68], as (Figure 2). Hence, in certain PDAC cancer types with deficiencies in homologous recombination, PARP inhibition is a promising strategy. Importantly, the Pancreatic Cancer Olaparib Ongoing (POLO) trial showed improved median survival and response rates in patients with germline BRCA mutations randomised to receive the PARP inhibitor Olaparib as maintenance therapy when compared to a placebo [69]. Although this does highlight some positive initial results, the use of a placebo and discontinuation of all other therapies in patients randomised to the control arm limits this work as this is not standard practice. Furthermore, given that only 14% of patients with PDAC have a BRCA or PALB2 mutation [12], these results are only applicable to a small subset of PDAC patients. However, two phase II studies evaluating the use of Olaparib in 46 previously treated PDAC patients with DDR deficiencies other than BRCA showed a response rate of 2%, a stable disease rate of 72%, and median overall survival of 9.9 months [70]. Given that such genetic alterations are found in a further 10–20% of PDAC patients [12], these results indicate that PARP inhibitors may be more widely therapeutically efficacious and could be a promising future direction in the field. Furthermore, novel approaches combining PARP inhibition with other agents could allow their use in a greater subset of patients. Although phase I studies combining PARP inhibition with alternative chemotherapy drugs had problems with toxicity (including grade 3+ treatment-related adverse events in >80% of patients) [71,72], a combination of PARP inhibitors with immune checkpoint inhibitors proved to be synergistic in mouse models of multiple solid tumours [73] and could be a useful alternative combination. Phase II trials combining niraparib and ipilimumab or nivolumab in PDAC patients responsive to platinum-based chemotherapy are ongoing [74]. 

ATR and ATM, serine/threonine kinases involved in DNA damage response (DDR) signalling [75], are other targets being investigated in this area. ATM loss, which promotes tumourigenesis through cell-cycle progression [76], is seen in 9–18% of patients with PDAC and predicts poor prognosis in those with loss-of-function mutations [77]. ATR is known to be involved in response to both single- and double-stranded DNA breaks and is critical in maintaining DNA integrity during replication [78]. Targeting ATM or ATR would hinder cellular DDR and thus evoke synthetic lethality, with research suggesting that ATR inhibitors may be particularly effective as the loss of ATM (prevalent in PDAC) leads to reliance on the ATR pathway for cellular survival following DNA damage [79]. ATM and ATR inhibitors are currently in the early stages of development for patients with solid tumours [78] and will hopefully progress to clinical trials.

## 5. Immunotherapies

**Figure 3:**
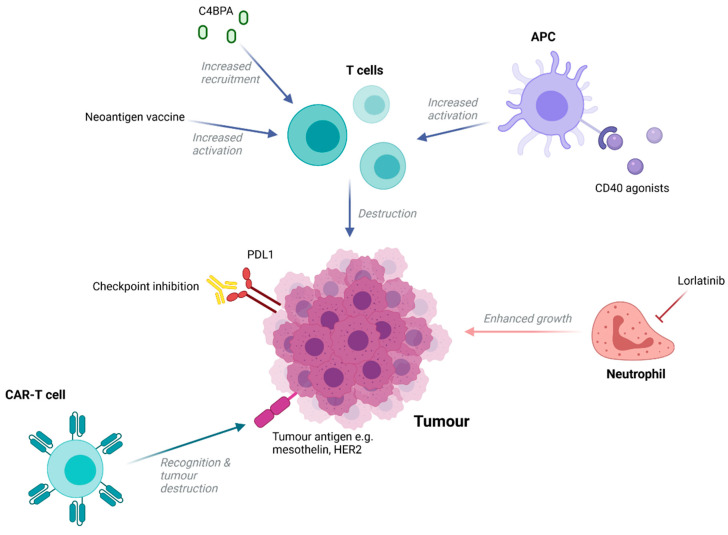
Immunotherapeutic targets in PDAC. Strategies in PDAC have focused heavily on enhancing T-cell-mediated immune responses, including through the use of CD40 agonists and neoantigen vaccines to enhance activation or application of molecules to increase T-cell recruitment (such as C4BPA). Checkpoint inhibitors also serve to enhance anti-tumour immune responses and, although their application as a single agent has proved ineffective, investigations into their efficacy in combination with other therapies continues. The introduction of CAR-T cells to promote tumour destruction has also been tested and despite disappointing initial results (a common theme amongst solid tumours), trials are ongoing. Newer research has involved targeting alternative immune cells, such as neutrophils, which are abundant in the tumour microenvironment and appear to promote tumour growth. This could be achieved through the application of the antibody Lorlatinib, which is able to suppress neutrophil accumulation, development, and activity.

Despite being widely regarded as an immunologically ‘cold’ cancer type there has been increasing interest in the potential of immunotherapies to treat PDAC (Figure 3). Disappointingly, single-checkpoint blockade therapies have been shown to be ineffective in treating patients [80,81], with resistance to this form of immunotherapy being multifactorial in nature. A lower mutational burden compared to malignancies known to respond to immune checkpoint inhibitors (ICIs) [82], an immunosuppressive microenvironment, and a dense desmoplastic stroma that act as a physical barrier to immune cell infiltration are all thought to be contributing factors [83]. However, it is possible to modulate these factors to increase immune-responsiveness and this has proven to be an attractive approach in recent research. 

For example, agonists to CD40, a member of the TNF receptor superfamily commonly expressed in immune cells, enabled antigen-presenting cells to increase levels of T-cell activation in tumour explants from a mouse KPC model [84]. A phase I trial in which an agonistic anti-CD40 antibody was administered in combination with nab-paclitaxel and gemcitabine with or without nivolumab demonstrated a 58% response rate amongst 24 patients [85], highlighting the potential of this approach. Alternatively, recent studies have shown that complement performs seminal functions in promoting or suppressing cancer progression. New research indicated that high levels of stromal C4b binding protein alpha chain (C4BPA) within human PDAC tissue were associated with better prognosis, including longer OS, and strongly correlated with increased levels of CD8+ tumour-infiltrating lymphocytes [86]. In vivo, GnP/ICBs/mC4BPA peptide treatment, but not GnP treatment, led to the accumulation of a greater number of CD8+ T cells in the periphery of PDAC tumours and increased tumour regression [86]. This highlights C4BPA stimulation as another potential method that could convert PDAC to an immunologically ‘hot’ tumour and thus increase the curative potential of ICIs. 

Outside of ICIs, multiple immunotherapies have been trialled with varying results. Mesothelin-targeting CAR-T cells have been trialled in six patients and were well tolerated, with one patient experiencing a 69% decrease in metabolically active tumour volume [87]. However, as with other solid tumours, lack of accessibility for circulating CAR-T cells and a rarity of uniformly expressed targetable epitopes is likely to hinder therapeutic efficacy. Nonetheless, additional trials involving CAR-T cells targeting mesothelin and other antigens are ongoing. Additionally, novel work using a specific subset of effector memory T-cells expressing CD161 as opposed to bulk T cells to generate HER2-specific CARs demonstrated the potential to improve CAR-T constructs in PDAC [88]. Importantly, the use of this particular T-cell population was able to enhance cytotoxicity and improve tumour burden control in mouse models of pancreatic cancer when compared to bulk peripheral mononuclear blood cells. 

Vaccine therapy has also been tested, with GVAX, an allogeneic whole-cell cancer vaccine, expressing a granulocyte macrophage colony stimulating factor, being used in combination with chemotherapy during the ECLISPE trial [89]. Disappointingly, GVAX failed to generate any improvement in OS, although increased T-cell recruitment to tumours was noted, suggesting that the vaccine may be effective in combination with ICIs to enhance anti-tumour immune responses. Additionally, PancVAX, a neoantigen-targeted vaccine, has been investigated in combination with a STING agonist in cell-line-derived PDAC xenografts and was able to demonstrate modest levels of activity [90]. 

New research has also aimed to target immune cell types outside of T cells. Neutrophils are abundant within the PDAC TME and are associated with poor clinical prognoses. They are known to promote tumour growth through ECM modulation and cytokine production and are also thought to aid in metastasis by supporting the survival of circulating cancer cells, enhancing stem cell characteristics of metastasis initiating cells, and establishing a hospitable niche for metastasis growth [91,92,93]. This highlights neutrophils as a promising target, with their inhibition being able to prevent both cancer growth and spread. Lorlatinib, a small molecular tyrosine kinase inhibitor able to suppress neutrophil accumulation, development, and activity, was able to attenuate PDAC progression and growth at both primary and metastatic sites in a PDAC mouse model [94]. Furthermore, when combined with an anti-PD-1 blockade, lorlatinib increased the levels of activated intra-tumoural CD8+ T cells and resulted in significantly smaller tumours compared to control and monotherapy groups [94].

## 6. Extracellular Tumour Microenvironment Modification

The abundance of fibrotic stroma, known as desmoplasia and derived mainly from pancreatic stellate cells, is a feature typical of PDAC. This dense extracellular matrix acts as a physical barrier preventing delivery of therapeutic agents to tumours and barring entry of immune cells to contribute to the ‘immunologically cold’ TME [95,96,97]. Hence, approaches that target the tumour stroma in addition to directly targeting PDAC cells are attractive. One strategy involved targeting focal adhesion kinase (FAK), a tyrosine kinase that promotes tumour invasion, growth, and metastasis through interactions with stromal cells in a number of solid malignancies [98]. In a KPC mouse model of PDAC, FAK inhibition reduced fibrosis and improved survival [96], with clinical trials investigating combined FAK inhibition and ICIs or cytotoxic agents now underway. Furthermore, positive results have been observed upon targeting connective tissue growth factor (CTGF), which is highly expressed in preclinical models of PDAC and is thought to contribute to desmoplasia [99]. Pamrevlumab, an anti-CTGF monoclonal antibody, led to improved OS in PDAC patients in combination with standard chemotherapy in a phase I/II trial [100]. On the basis of these results, pamrevlumab has now been designated as a fast-track therapy to treat patients with locally advanced PDAC. However, there is evidence that suggests that components of the stroma may act to restrain cancer cell growth and impede metastasis [101], with reports that poorly differentiated PDAC with low desmoplasia is more aggressive and has a worse prognosis [102]. Thus, caution must be taken when targeting the stroma in order to ensure that the effects are beneficial. 

This is illustrated in the case of targeting hyaluronic acid (HA), a glycosaminoglycan (GAG) overexpressed in PDAC stroma and associated with low immune response and poor prognosis [103,104]. HA was initially thought to be a promising target in PDAC, with one study involving HA staining on samples from 101 patients with stage IA-IIB disease revealing that high levels of stroma HA expression were significantly associated with poor disease specific survival and OS [105]. In mouse models, treatment with PEGPH20 (a pegylated recombinant human hyaluronidase that breaks down hyaluronan) and gemcitabine prolonged survival [106], while a phase II trial administering PEGPH20 and nab-paclitaxel/gemcitabine to patients with advanced-stage PDAC found that the addition of PEGPH20 significantly improved PFS in patients with high levels of hyaluronic acid expression [107]. However, despite these early encouraging results, a phase III trial (HALO-301) involving the administration of PEGPH20 plus nab-paclitaxel/gemcitabine (PAG) or placebo plus nab-paclitaxel/gemcitabine (AG) failed to meet its primary endpoints [108]. Disappointingly, OS in the PAG group was slightly lower than the AG group (11.2 months vs. 11.7 months) and there was no difference in PFS between the two. Furthermore, results from a phase Ib/II trial in which PEGPH20 was combined with mFOLFIRINOX in patients with metastatic PDAC demonstrated that PEGPH20 addition was detrimental to OS [109], re-iterating the potential adverse consequences of stromal targeting. Alternatively, trial failure could have resulted from the targeting of desmoplasia alone being insufficient to evoke treatment benefits with other factors such as low tumour mutational burden, lack of targetable neoantigens, and EMT also requiring modulation to produce favourable results [110].

## 7. Elimination of Cancer Stem Cells 

Cancer stem cells (CSCs) refer to a specific subset of cells within tumours, which display self-renewal and the ability to produce differentiated progeny, characteristics typically associated with normal stem cells. First described in 1997 in acute myeloid leukaemia [111], CSCs have since been discovered in multiple other cancer types, with the first report of their presence in PDAC arising in 2007 [112]. Since then, pancreatic CSCs (PCSCs) have been shown to play a vital role in PDAC propagation and growth [113]. More recently, they have been linked to tumour stroma differentiation, with a greater presence of PCSC markers being associated with a loose stroma type and higher rates of cumulative local recurrence [114]. Furthermore, PCSCs are known to contribute to chemoresistance in PDAC [115] and cancer recurrence following treatment, hence curing PDAC is contingent on complete CSC annihilation. 

Much work has been carried out in this area aiming to characterise and selectively target pancreatic CSCs. JAK-STAT and Hedgehog pathways, known to be involved in CSC maintenance [116,117], have been targeted in PDAC but failed to produce satisfactory results [118], highlighting a need for better characterisation of CSC signalling. Alternatively, it is possible to target specific surface markers of CSCs, which include CD44, CD24, CD133, CXCR4, and ESA [112,113]. For example, the generation of a bi-specific antibody recognising ESA and CD3 was able to re-direct cytotoxic T lymphocytes to eliminate highly tumourigenic PCSCs in vitro and in vivo [119]. 

Specific metabolic targeting of CSCs is also a promising approach, with PCSCs highlighted as being dependent on oxidative phosphorylation in (OXPHOS) in order to survive. Inhibition of this pathway by metformin was able to produce rapid apoptosis specifically within the PDAC CSC population while non-CSC cells were less dramatically affected, only undergoing cell cycle arrest [120]. Furthermore, recent work on four PDAC cell lines has shown that PCSCs have specific and common proteome and lipidome modulations, which could prove to be useful therapeutic targets [121]. These include mitochondrial cardiolipin acyl chain modification and upregulated fatty acid elongation and phosphoinositol phosphatase pathways, features unique to PCSCs and possible targets for drug development.

**Figure 4:**
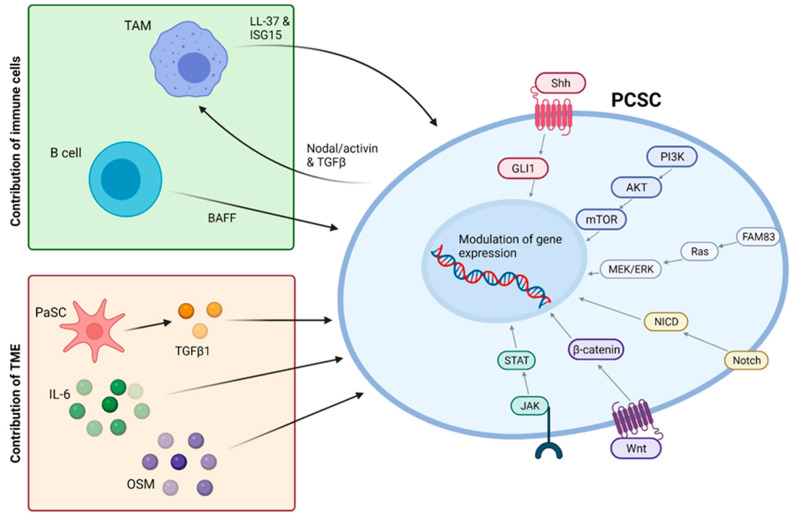
Pathways promoting cellular plasticity in PCSCs. Top left: Immune cells are known to play an important role in CSC plasticity, with the production of B-cell activating factor (BAFF) by infiltrating B lymphocytes inducing EMT and enhancing the motility of pancreatic epithelial cells. Tumour-associated macrophages (TAMs) secrete the immune modulatory peptide LL-37, which enhances stemness and sphere formation. The secretion of Nodal/Activin and TGFβ-1 by CSCs stimulates further LL-37 secretion to generate a positive feedback loop. TAMs also secrete interferon-stimulated gene 15 (ISG-15), which enhances CSC self-renewal, pluripotency, and invasion. Bottom left: The tumour microenvironment (TME) influences CSC emergence, with the addition of TME cytokines to tumour cells resulting in the acquisition of stem cell characteristics. Transforming growth factor beta (TGFβ), interleukin 6 (IL-6), and oncostatin (OSM) all induce mesenchymal/CSC properties. Pancreatic stellate cells (PaSCs) are known to induce CSC properties in neighbouring cells, which could be mediated in part by the production of TGFβ. Right: Multiple signalling pathways evoke plasticity, including mTOR, sonic hedgehog (Shh), Notch, Wnt, and Stat. Family with Sequence Similarity 83 (FAM83) proteins have been shown to drive cellular transformation, with FAM83A driving PDAC cell survival via the MEK/ERK signalling pathway.

However, it is important to note that increasing bodies of work suggest that PCSCs, and CSCs as a whole, are not a defined subpopulation but rather a state that non-CSC tumour cells are also able to enter [122]. This means that not only is it important to target existing CSCs but also to understand and inhibit pathways promoting cellular plasticity that may give rise to new CSCs. Such pathways have been extensively reviewed by Smigiel et al. (2018) [118] and are summarised in Figure 4. Many of these targets have already demonstrated promising pre-clinical results, with combined targeting of mTOR and c-MET signalling eliciting a dramatic reduction in the viability of CD133+ CSCs while the inhibition of Notch signalling by genistein significantly reduced self-renewal of PCSCs [118]. Furthermore, pharmacological inhibition or knockdown of ALK4, the target receptor of Nodal and Activin, demonstrated the ability to abolish the self-renewal and tumourigenicity of PCSCs and sensitised this subpopulation to gemcitabine [123]. 

## 8. Targeting Epigenetic Mechanisms

**Figure 5:**
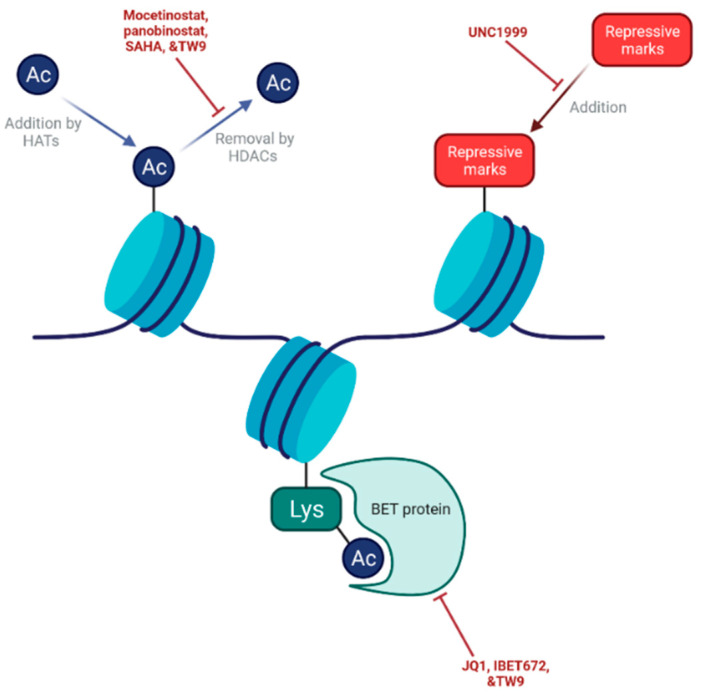
Epigenetic targets and drugs in PDAC. Much research has focused on histone deacetylase (HDAC) inhibitors, which have the potential to abolish cellular plasticity through the reversal of epigenetic states. However, both mocetinostat and Panobinostat failed in clinical trials. Bromodomain and extra-terminal domain (BET) proteins, which play key roles in the modulation of gene expression through binding to acetylated lysine residues, are alternative targets. In vitro and in vivo pre-clinical models have demonstrated the efficacy of BET inhibitors I-BET 627 and JQ1. UNC1999, an inhibitor of writers for H3K9me2 and H3K7me3 repressive markers, has also achieved promising results in organoid models.

Alteration to epigenetic pathways within cells is known to be vital in cancer onset and progression and is thought to stimulate stem cell-like characteristics within tumour cells. Targeting epigenetic mechanisms in order to abolish this cellular plasticity and trap cells in a more restricted state, thus re-sensitising tumours to chemotherapy, is a promising approach to ablate PCSCs (Figure 5). Histone deacetylase (HDAC) enzymes, which modulate gene expression through histone modification, are key players in epigenetic processes and their inhibition can reverse epigenetic states [124]. HDAC inhibitors have already displayed promising results in haematological malignancies and have been approved by the Food and Drug Administration in the USA for the treatment of cutaneous T-cell lymphoma [125]. However, two HDAC inhibitors, mocetinostat and Panobinostat, have both failed in Phase II trials in PDAC patients [126,127], suggesting that alternative epigenetic targets are needed. 

This could take the form of bromodomain and extra-terminal domain (BET) proteins, which are able to bind to acetylated lysine residues on histones and subsequently modulate gene expression. Promisingly, in PDAC, the bromodomain inhibitor I-BET 672 showed anti-tumour activity and cytotoxic synergy with gemcitabine in vitro and in vivo in pre-clinical research [128,129]. Furthermore, the BET inhibitor JQ1 was able to demonstrate efficacy in both 3D collagen [130] and xenograft [131] models of PDAC, blocking cancer cell growth in both. More recently, JQ1 demonstrated synergy with the PARP inhibitor Olaparib [132] and anti-PD-L1 antibodies [133] in pre-clinical models to evoke improved inhibition of tumour growth, highlighting the potential to include BET inhibitors in combination with other agents to enhance efficacy. JQ1 also synergised with the HDAC inhibitor SAHA to augment cell death in vitro and in vivo [134], which highlights the novel compound TW9, a potent dual inhibitor of both HDAC and BET proteins [135], as a particularly promising development. 

A further positive step forward has arisen from new models, which will enable clinicians to better tailor epigenetic therapy regimes to specific patients. Organoids, small 3D multicellular in vitro constructs able to mimic in vivo structures, have proven to be capable of recapitulating primary tumour characteristics in PDAC and modelling unique patient cancers [136,137]. Importantly, drug screening was able to highlight distinctive sensitivities in many patient cancers [136], which could enable improved treatment and outcomes. Huang et al. were able to highlight the compounds A366 and UNC1999, inhibitors of writers for H3K9me2 and H3K7me3 repressive markers, respectively, as candidates for novel epigenetic drugs through screening on five tumour organoid models [137]. UNC1999 then went on to demonstrate dose-dependent decreases in cellular proliferation when combined with gemcitabine in five out of six organoids tested [137], confirming its potential as a novel agent. 

More recently, Bian et al. were able to define MYC-high and MYC-low PDAC subtypes using a set of 16 transcriptional targets of c-MYC in patient-derived tumour xenograft (PDTX) models, with the MYC-high subgroup demonstrating increased sensitivity to the BET inhibitor JQ1 [138]. This information could be useful in guiding clinical decision making but is unlikely to be practical in PDAC given the timeframe of 6–8 months required. However, MYC-high and MYC-low subtypes were able to be recapitulated in patient-derived organoids, with MYC-high organoids displaying a similar sensitivity to BET inhibitors as seen in PDTX models [139]. Given the much shorter timeframe of just 2–3 weeks needed to generate enough material for organoid analysis, this work highlights organoids as a useful model to guide personalised epigenetic treatments. 

## 9. Conclusions

Despite only modest improvements in patient outcomes, there have been multiple steps forward in PDAC treatment over the past few years. Importantly, mFOLFIRINOX has been highlighted as the standard-of-care adjuvant therapy, and combined chemotherapies such as paclitaxel and gemcitabine or FOLFIRINOX have been developed for advanced-stage disease. Furthermore, Olaparib maintenance therapy has been shown to improve outcomes in those with BRCA mutations and ICIs could enable long-term survival in patients with MMR deficient PDAC. 

As with oncology as a whole, in recent years, PDAC literature has demonstrated a clear shift from classical chemotherapy to more targeted approaches. This has led to a greater emphasis on strategies such as pathway inhibition, metabolic targeting, and attack of PCSCs. Unfortunately, there are multiple challenges affecting the development and application of precision medicine in PDAC, including rapid disease progression requiring a short timeframe between diagnosis and commencing treatment, the difficulty of tissue acquisition for tumour samples, and tumour heterogeneity, which is likely to limit specific pathway inhibitors to small subsets of patients [23]. Despite this, there have been promising steps forward in the field, most notably the direct inhibition of the G12C mutant variant KRAS. If this work could be expanded to produce inhibitors able to target highly prevalent KRAS mutations, it would revolutionise PDAC treatment. 

Outside of pathway inhibition, immunotherapeutic, stromal, and epigenetic approaches have demonstrated encouraging results and are set to become more prominent. Methods aiming to convert PDAC from an immune ‘cold’ to an immune ‘hot’ tumour could open up the disease to whole new areas of medicine through enabling immunotherapeutic targeting. Meanwhile, targeted therapies and epigenetic approaches show promise in the annihilation of PCSCs, known to be a prerequisite for the successful treatment of PDAC. These promising results over several areas of research will hopefully translate to improved patient outcomes in the coming years.

## Figures and Tables

**Table 1:** PDAC subtypes.

Classification	Subtype	Characteristics
Collison	Classical	Better prognosis than QM subtype following resection High expression of adhesion-associated and epithelial genes, high GATA6 expression Increased dependence on KRAS and increased sensitivity to erlotinib compared to QM subtype
	Quasi-mesenchymal (QM)	High expression of mesenchyme associated genes Increased sensitivity to gemcitabine compared to classical subtype
	Exocrine-like	Relatively high expression of tumour cell-derived digestive enzyme genes
Moffitt tumour-specific subtypes	Classical	20/22 genes shared with Collisson classical subtype
	Basal-like	Worse median survival and 1-year survival compared to basal-like subtype Better response to adjuvant therapy compared to classical subtype High expression of laminins and keratins
Moffitt stromal-specific subtypes	Normal	High expression of markers for pancreatic stellate cells, smooth muscle actin, vimentin, and desmin
	Activated	Worse median and 1-year survival compared to normal subtype More diverse gene expression including genes associated with macrophages (e.g., *ITGAM*, *CCL13*, and CCL18) and genes involved in tumour promotion (e.g., *SPARC*, *WNT2*, *WNT5A*, *MMP9*)
Bailey	Squamous	Poor prognosis compared to other subtypes High expression of TP53 and KDM6A, and upregulation of TP63∆N transcriptional network Hypermethylation of pancreatic endodermal cell-fate determining genes
	Pancreatic progenitor	High expression of genes involved in early pancreatic development (*FOXA2/3*, *PDX1*, and *MNX1*)
	Immunogenic	Significant immune infiltrate Upregulation of immune networks, including pathways associated with acquired immune suppression
	Aberrantly differentiated endocrine exocrine	Subclass of pancreatic progenitor tumours High expression of genes involved in regulating KRAS activation, exocrine (*NR5A2* and *RBPJL*), and endocrine differentiation (*NEUROD1* and *NKX2-2*)

**Table 2:** Pathway inhibition strategies in PDAC.

Pathway	Function	Relevant Results
Growth factor receptors and associated pathways		
Epidermal growth factor (EGF) pathway	Important regulator of cell survival, able to activate multiple signalling pathways including PI3K/Akt, Ras/Raf/MEK/ERK, and Jak-STAT. EGF receptor (EGFR) is essential in PDAC tumourigenesis as its activity is needed for KRAS activation of ERK [24].	Addition of the EGFR inhibitor erlotinib to gemcitabine significantly prolonged OS compared to gemcitabine alone in a phase III trial [25]. More recently, combined inhibition of EGFR and c-RAF induced complete tumour regression in genetically engineered PDAC mouse models and blocked tumour progression in patient-derived xenografts harbouring KRAS and TP53 mutations [26].
KRAS	KRAS mutations are present in >90% of PDACs [20] and are thought to drive PDAC initiation [27].	KRAS^G12C^ mutant inhibitors were able to inhibit tumour growth in vivo in pre-clinical models [13]. In phase I trials in patients with lung adenocarcinoma or colorectal cancer, the KRAS^G12C^ inhibitor AMG-510 was well tolerated and able to induce stable disease or partial response [14,28].
Ras/Raf/MEK/ERK	Mediates cellular response to several growth factors.	Gemcitabine and pimasertib (MEK inhibitor) showed synergistic inhibition of tumour growth in PDAC mouse models [29].
NRTK	Fusion of TRKA, TRKB, or TRKC proteins with a variety of partners leads to constitutive activation of Ras/Raf/MEK/ERK pathway and PI3K/AKR pathway to aid in tumourigenesis [30].	In the NAVIGATE trial, selective TRK inhibitor Larotrectinib was able to produce a 75% response rate in patients with NRTK-fusion positive tumours, including one patient with PDAC who had a partial response [31] The STARTRK-2 trial investigated the use of entrectinib (an inhibitor of TRKA/B/C and Ros-1 or Alk-containing gene fusions) in 3 patients with PDAC and demonstrated clinical improvement in all 3 [32].
Evading growth suppressors		
Aurora kinase	Aurora kinase proteins play important roles in cell division and are overexpressed in a number of tumours.	Danusertib (pan-Aurora kinase inhibitor) showed limited clinical activity as a second line treatment in PDAC with just 3 out of 31 patients remaining stable for 6–8.5 months [33].
Cyclin dependent kinase (CDK)	CDK proteins, overactivated in cancer, are vital cell cycle regulators and play a role in controlling RNA transcription. Loss of CDKN2A is frequently seen in PDAC and detected in 47% of patients in one study [21].	One mouse xenograft study showed that CDK4/6 inhibitors prevent PDAC recovery following taxane-based chemotherapy [34]. However, clinical trials of several CDK inhibitors have failed to show the desired results in PDAC and solid tumours as a whole [35].
Angiogenesis inhibitors		
Vascular endothelial growth factor (VEGF)	VEGF pathway is a key regulator of angiogenesis and vital in enabling the development of a blood supply to support tumour growth.	Numerous clinical trials of VEGF inhibitors in PDAC have failed to show clinical benefit, with phase III trials combining gemcitabine with bevacizumab or axitinib failing to reach their primary endpoints of OS [36]. However, one study assessing the combination of the VEGF inhibitor bevacizumab with 5-FU, leucovorin, nab-paclitaxel, and oxaliplatin did report an impressive OS of >17 months [37].
Invasion and metastasis inhibitors		
TGF beta	TGF-beta pathway plays a role in cell proliferation, differentiation, migration, and apoptosis. Recently, it has been shown to switch from an initial anti-tumourigenic role to a pro-tumourigenic role during the course of cancer progression [38].	The TGF beta inhibitor galunisertib has shown promising results in combination with gemcitabine in PDAC, improving OS and PFS compared to gemcitabine alone [39,40].
Inhibitors of apoptosis resistance		
Insulin-like growth factor (IGF)	The IGF pathway stimulates cell proliferation, suppresses programmed cell death, and promotes cell differentiation. It has also been shown to aid in CSC maintenance and self-renewal in PDAC [41].	Dalotuzumab (an IGF-1R antagonist) improved OS but not PFS in combination with gemcitabine and erlotinib [42].
NF-KB	NF-KB suppresses apoptosis, downregulates p53 expression, and induces matrix metalloprotease expression to contribute to cancer growth and metastasis.	Curcumin and Theracurmin (NF-KB inhibitors) have been highlighted as promising options in PDAC [43] and combined Theracurmin and gemcitabine was shown to be well tolerated in one phase I trial [44].
Anti-inflammatory drugs		
JAK-STAT	This pathway is known to play a key role in cell proliferation, angiogenesis, and apoptosis [45], while STAT3 has been linked to the emergence of CSCs from non-stem cancer cells [46].	A phase III trial investigating the use of combined ruxolitinib (a specific JAK1/2 inhibitor) and capecitabine in patients with advanced/metastatic PDAC was terminated due to failure to improve OS [47]. This was similarly true for a phase I trial administering the JAK1 inhibitor momelotenib to patients with metastatic PDAC [48]. However, clinical trials into alternative JAK/STAT inhibitors are ongoing [49].
Src	Src deregulation is thought to enhance tumour growth and invasion.	The Src inhibitor Dasatinib failed in phase II trials for PDAC, both as a monotherapy [50] and in combination with gemcitabine [51].
Bruton tyrosine kinase (BTK)	BTK is a non-receptor tyrosine kinase of the B cell receptor signalling pathway.	BTK inhibition with ibrutinib was shown to restore the T-cell-driven anti-tumour response in mouse models of PDAC and enhance responses to chemotherapy [52]. However, in the RESOLVE trial, a phase III study investigating the addition of ibrutinib to nab-paclitaxel and gemcitabine, no benefit in OS or PFS was seen in patients with advanced-stage PDAC [53]. Acalabrutinib, an alternative BTK inhibitor, also showed limited clinical activity as a monotherapy or in combination with pembrolizumab [54].
